# Supplementary material for: A Critical View of the Application of the APEX Software (Aqueous Photochemistry of Environmentally-Occurring Xenobiotics) to Predict Photoreaction Kinetics in Surface Freshwaters
Source: Molecules. 2019 Dec 18;25(1):9. doi: 10.3390/molecules25010009 (PMC7017383; doi:10.3390/molecules25010009)
Supplement: Supplementary file 1 [file molecules-25-00009-s001.zip › 3.2.4_gcc-4.4.0.zip!/3.2.4_gcc-4.4.0/3.2.4_gcc-4.4.0/doc/octave/html/interpreter/Customizing-readline.html]

Customizing readline - Untitled


Next: Customizing the Prompt,
Previous: Commands For History,
Up: Command Line Editing


---

#### 2.4.6 Customizing `readline`

Octave uses the GNU Readline library for command-line editing and
history features. Readline is very flexible and can be modified through
a configuration file of commands (See the GNU Readline library for the
exact command syntax). The default configuration file is normally
~/.inputrc.

Octave provides two commands for initializing Readline and thereby changing
the command line behavior.

— Built-in Function: **read\_readline\_init\_file** (file)  
> Read the readline library initialization file file. If
> file is omitted, read the default initialization file (normally
> ~/.inputrc).
>
> See Readline Init File (GNU Readline Library),
> for details.

— Built-in Function: **re\_read\_readline\_init\_file** ()  
> Re-read the last readline library initialization file that was read.
> See Readline Init File (GNU Readline Library),
> for details.
